# 工程化外泌体在肺癌治疗中的研究进展

**DOI:** 10.3779/j.issn.1009-3419.2024.101.17

**Published:** 2024-07-20

**Authors:** Wanling YANG, Yan GU

**Affiliations:** 010050 呼和浩特，内蒙古医科大学附属医院; Affiliated Hospital of Inner Mongolia Medical University, Hohhot 010050, China

**Keywords:** 工程化外泌体, 靶向递送, 肺肿瘤, 基因治疗, 肿瘤微环境, Engineered exosomes, Targeted delivery, Lung neoplasms, Gene therapy, Tumor microenvironment

## Abstract

非小细胞肺癌最佳的治疗方式为早期手术治疗，而大部分肺癌发现时已为晚期。治疗方式以药物及放射治疗为主。但上述治疗方式出现耐药或疗效不显著是不可避免的，因此，针对肺癌的治疗迫切需要更多的手段。研究证实，工程化外泌体在心血管系统疾病、肿瘤、组织再生与修复等领域具有良好的临床应用潜能。本文总结了国内外工程化外泌体在肺癌治疗中的应用。

## Abstract (en)

The best treatment for non-small cell lung cancer is early surgical treatment, but most lung cancer is diagnosed at an advanced stage. The main treatment methods are drug and radiotherapy. However, drug resistance or no signifi cant effect of the above treatment methods is inevitable. Therefore, more methods are urgently needed for the treatment of lung cancer. Studies have confirmed that engineered exosomes have good clinical application potential in cardiovascular diseases, tumors, tissue regeneration and repair. This paper summarizes the application of engineered exosomes in the treatment of lung cancer at home and abroad.

据统计^[1]^，2018年全球死于肺癌的患者约176万，其中非小细胞肺癌（non-small cell lung cancer, NSCLC）约占85%。虽然靶向治疗、免疫检查点抑制剂以及多种方法的联合为晚期NSCLC患者带来了新的治疗希望，但NSCLC患者的总体预后仍然较差。外泌体是由细胞分泌的膜性囊泡，直径为40-160 nm^[2]^。它广泛存在于巨噬细胞、树突状细胞（dendritic cell, DC）、T细胞、肿瘤细胞、B细胞、上皮细胞、网织红细胞、肥大细胞、神经元等，以及哺乳动物细胞的外泌体细胞和施万细胞，在唾液、尿液、腹水等生物体液中也有较多的分泌，并在肿瘤的诊断、治疗方面拥有巨大的临床应用潜力^[3,4]^。外泌体具有改造性强、生物可降解性好、免疫原性低、生物相容性高等特性^[5,6]^。但天然外泌体容易在非特异性组织中潴留（如肝、脾），且装载药物能力有限，因此我们需要外泌体具有靶向性，可装载更多的内容物，能避免免疫系统清除，使外泌体具有更强的改善肿瘤微环境（tumor microenvironment, TME）的作用，以便满足临床需要，为此对外泌体进行生物工程技术内部或表面的改造和修饰，或使用细胞工程的方法对亲代细胞进行改造，得到的外泌体称为工程化外泌体。其中外泌体工程包括药物共孵育、皂化、物理法、表面修饰等。细胞工程包括细胞培养条件的改变、细胞培养中添加药物、基因工程。工程化外泌体已在心血管系统疾病、肿瘤、组织再生与修复等领域被证实具有良好的临床应用潜能，且与天然外泌体相比拥有更优秀的靶向能力和疗效。在肺癌的治疗方面，其可能为患者带来获益。本文综述了现阶段工程化外泌体在肺癌治疗中的研究进展。

## 1 工程化外泌体在肺癌化疗药物中的应用

目前NSCLC化疗药物有培美曲塞、白蛋白结合型紫杉醇、吉西他滨等。但无论哪种药物均有最终耐药的情况。化疗耐药的机制包括药物外排增多、药物靶点改变、细胞死亡异常、药物失活等。通过物理制备法、皂化、药物共孵育、表面修饰等可增加化疗药物在外泌体的装载能力，可增加药物在肿瘤部位的浓度。已有研究^[7]^表明，形成的工程化外泌体可携带药物起到抗肿瘤作用，如巨噬细胞来源的工程化外泌体通过装载紫杉醇等起到抗肿瘤的作用。Kim等^[7]^研究表明，将巨噬细胞的外泌体修饰在聚乙二醇（polyethylencglycol, PEG）的冠状结构中，目的是降低其免疫原性，同时装载氨基乙酰胺（amino acetamide, AA）以靶向NSCLC高表达的σ受体形成工程化外泌体，其与紫杉醇结合，结果显示这种装载紫杉醇的AAPEG外泌体具有更高的生物利用度、药物装载能力，可在肿瘤细胞内富集，具有更长的体内循环时间。全身给药时，这种装载紫杉醇的AAPEG拥有更强的抗肿瘤作用，与肺癌转移灶的共定位率可达94.4%。

## 2 工程化外泌体在肺癌免疫治疗及TME中的应用

免疫检查点抑制剂的靶点主要有程序性死亡配体1（programmed cell death ligand 1, PD-L1）、细胞毒性T淋巴细胞抗原-4（cytotoxic T-lymphocyte antigen-4, CTLA-4）、程序性细胞死亡受体1（programmed cell death 1, PD-1），以及正在研究的跨膜蛋白淋巴细胞活化基因3、T淋巴细胞免疫球蛋白黏蛋白3、V域免疫球蛋白T细胞活化抑制因子等^[8,9]^。PD-1、PD-L1、CTLA-4相关抑制剂通过激活抗肿瘤T细胞来逆转免疫抑制状态，增强抗肿瘤免疫^[10]^。目前肺癌临床应用的免疫检查点抑制剂包括帕博丽珠单抗、阿替利珠单抗、西米普利单抗、替雷利珠单抗、信迪利单抗等。且帕博丽珠单抗、西米普利单抗、阿替利珠单抗被美国食品药品监督管理局（Food and Drug Administration, FDA）推荐用于驱动基因阴性的PD-L1高表达晚期NSCLC患者的一线治疗^[11]^。虽然免疫检查点抑制剂在肺癌治疗中起到了非常重要的作用，但部分患者存在免疫检查点治疗疗效不佳的情况，其原因可能与抑制性TME的发生有关^[11]^，为获得更好的抗肿瘤疗效，这种治疗手段需要克服药物的不稳定性、递送效率低、免疫相关不良反应和缺乏疗效等困难^[12]^。可利用工程化外泌体重塑TME。

M2型肿瘤相关巨噬细胞（tumor-associated macrophages, TAMs）是抑制性免疫细胞的主要亚群，与调节性T细胞参与TME的形成。通过工程化外泌体（或与其他疗法联合）将巨噬细胞从M2样表型重编程为M1样表型被认为是逆转TME的一种现实策略^[13⇓-15]^。信号转导和转录激活因子6（signal transducer and activator of transcription 6, STAT6）是控制M2表型的关键转录因子^[16]^。M1型巨噬细胞来源的外泌体负载靶向STAT6的反义寡核苷酸可单独诱导一氧化氮合酶2的表达，导致TME重塑，并激活CD8 T细胞介导的适应性免疫应答^[13]^。此外，环磷酸鸟苷-腺苷酸合成酶/干扰素基因刺激因子（cyclic guanosine monophosphate-adenosine monophosphate synthase/stimulator of interferon gene, cGAS/STING）介导的固有免疫在肿瘤治疗中显示出良好的应用前景。激活STING通路可以促进DC的成熟，促进肿瘤细胞毒性T淋巴细胞（cytotoxic T lymphocytes, CTL）和自然杀伤（natural killer, NK）细胞浸润。Cheng等^[17]^将携带CD47的肿瘤细胞工程化外泌体和M1型巨噬细胞的外泌体融合，设计了用于cGAS/STING激活的多功能混合外泌体，并进一步用DNA靶向剂（SN38）和STING激动剂（MnO_2_）包裹这些外泌体，形成的杂交外泌体显示出了良好的肿瘤靶向性，并延长了其在血液循环中的时间。在肿瘤部位，杂交外泌体诱导TAMs向M1表型极化并释放SN38诱导DNA损伤和Mn^2+^刺激cGAS/STING激活并促进DC成熟，促进CTL浸润和NK细胞募集到肿瘤区域，从而产生显著的抗肿瘤和抗转移效果^[17]^。在一项针对NSCLC患者化疗停止4个月后的维持性免疫治疗的II期临床试验^[18]^中，对装载有主要组织相容性复合体（major histocompatibility complex, MHC）分子限制性肿瘤抗原的成熟DC的外泌体进行疗效评估，其中位生存时间为15个月，中位无进展生存时间为2.2个月，仅有1例发生3级肝毒性。

表达成纤维细胞活化蛋白（fibroblast activation protein, FAP）-α的外泌体可作为肿瘤疫苗，诱导增强针对肿瘤细胞和FAP+癌症相关成纤维细胞（cancer associated fibroblasts, CAFs）的CTL免疫应答^[19]^。这些基于外泌体的肿瘤疫苗可重塑结直肠癌、黑色素瘤、肺癌等的TME^[19]^。FAP+CAFs也可作为工程化外泌体重塑TME的靶点，靶向FAP基因的肿瘤来源的外泌体样纳米囊泡可以在多种模型中触发针对肿瘤细胞和FAP+CAFs的强效CTL免疫应答，其激活的免疫应答可通过从CTL释放γ干扰素和消耗FAP+CAFs来启动肿瘤铁死亡^[20]^。诱导肿瘤坏死因子凋亡相关配体（tumor necrosis factor-related apoptosis-inducing ligand, TRAIL）可选择性诱导肿瘤细胞凋亡，但其临床应用受到其低生物利用度和肿瘤细胞耐药的限制。利用工程化的间充质干细胞（mesenchymal stem cells, MSCs）产生含有TRAIL的外泌体，可改善这一弊端，它不仅可导致包括乳腺、肾、肺和间皮瘤多种癌细胞系的凋亡，还能抑制肿瘤的转移^[21]^。

上述研究表明，通过工程外泌体改善TME是抗肿瘤免疫治疗中的一种切实可行的策略，有很大的研究前景。

## 3 工程化外泌体在肺癌基因治疗中的应用

基因治疗已被证明是治疗NSCLC的一种有前景的方法，简单地定义为通过基因修饰细胞来产生治疗效果^[22]^。基因治疗的方法一种是将特定DNA片段通过基因编辑进行删除和添加。另一种是将外源基因导入细胞切割缺陷基因mRNA转录物中的特定序列，阻止翻译缺陷mRNA，从而达到基因治疗的目的^[23]^。近年来，已开发出多种用于递送核酸的方法，主要包括病毒载体和非病毒载体^[24,25]^。病毒载体具有毒性和免疫原性，而非病毒载体转导效率低，粒径较大，容易诱导免疫应答。因此可使用基因递送载体解决上述问题，目前微小RNA（microRNA, miRNA）和小干扰RNA（small interfering RNA, siRNA）常用于基因治疗。多种miRNA在体内可稳定存在^[26]^，并可以转移到体内的其他细胞和器官，在新的部位产生不同的效果，同时可以调控肺癌的增殖、凋亡和化疗敏感性^[27⇓⇓⇓⇓-32]^。

有研究^[33]^选择肺癌中表达下调的miR-563作为靶miRNA，形成负载miR-563的工程化外泌体，使用其处理A549细胞，可观察到肿瘤伤口闭合明显延迟，与对照组相比，该工程化外泌体处理的肺癌细胞侵袭和迁移能力明显受到抑制。在模型小鼠中很容易发现装载miR-563的外泌体示踪染料标记的工程化外泌体。与对照组相比装载miR-563的工程化外泌体组小鼠的肿瘤体积明显减小，肿瘤重量显著降低，且细胞凋亡水平增加^[33]^。检测谷草转氨酶、碱性磷酸酶、谷丙转氨酶、血常规等提示miR-563负载的工程外泌体不影响肝功能、红细胞、白细胞、血小板计数。对心脏、肝脏、脾脏、肺脏和肾脏染色显示这些器官均未出现组织病理学异常。体内实验评估miR-563工程化外泌体的抑瘤效果与未负载miR-563的外泌体相比，制备的工程化外泌体处理的小鼠肿瘤体积和重量明显缩小^[33]^。

细胞归巢穿模肽（truncated LYP-1, tLyp-1）是一种7肽（氨基酸序列CGNKRTR），可穿透肿瘤血管和间质，并靶向肿瘤，是选择性靶向神经纤毛蛋白1（neuropilin 1, NRP1）和NRP2的配体，这两种蛋白在肿瘤组织中高表达，包括可在NSCLC中高表达，并可作为肿瘤药物传递系统的受体靶点^[34⇓-36]^。有研究^[37]^构建了工程化的tLyp-1-溶酶体相关膜蛋白2b（tLyp-1-lamp2b）质粒。扩增后将该质粒转染到HEK293T细胞，再从转染后的细胞分泌物中分离靶向tLyp-1外泌体，并将人工合成的siRNA利用电穿孔技术包裹到靶向tLyp-1外泌体中，利用靶向siRNA tLyp-1外泌体转染肿瘤细胞或肿瘤干细胞。结果显示，这种构建的靶向tLyp-1外泌体对肿瘤干细胞和肿瘤细胞均具有较高的转染效率，能够敲低肿瘤细胞的靶基因，并降低肿瘤干细胞的“干性”。

在NSCLC细胞或组织中，miR-21、miR-574-5p、miR-26、miR-155、miR-1254、miR-449a等miRNAs通过细胞凋亡、逃逸、细胞周期调节、浸润转移调节、炎症调节等方面影响NSCLC的进展^[38⇓⇓⇓⇓-43]^。因此，在常规化疗药物治疗过程中纠正NSCLC细胞中失调的miRNA可增加传统化疗药物的敏感性。为此Nie等^[44]^制备了一种递送miRNA-126的肺靶向外泌体。这种工程化外泌体有效地抑制了A549细胞的增殖和转移，并在裸鼠模型中表现出抗癌作用，结果表明外泌体可作为高效载体携带特定治疗基因用于包括NSCLC在内的疾病的基因治疗。利用基因工程方法制备能够主动递送miR-449a的工程化外泌体^[45]^。与miR-449a工程化外泌体共孵育72 h后，A549细胞的活力明显降低，未链接miR-449a的外泌体、PBS外泌体对A549细胞活力几乎没有影响。miR-449a工程化外泌体处理组的凋亡率明显高于其他对照组，miR-449a工程化外泌体处理组A549细胞中miR-449a的表达量最高，与其他组有显著差异。同时，miR-449a工程化外泌体具有良好的同源靶向能力和较高的递送效率，且可明显下调A549细胞中抑制细胞凋亡的基因B细胞淋巴瘤2（B-cell lymphoma/leukemia 2, Bcl-2）的表达，最终调控肿瘤的进展^[45]^。另一项研究^[46]^显示，携带KRAS基因特异度的siRNA的外泌体对A549细胞可产生剂量依赖性的抗肿瘤增殖效应，而且在小鼠肺癌模型中，携带siKRAS的叶酸化的外泌体显著抑制肿瘤生长。

## 4 工程化外泌体在肺癌放疗中的应用

放疗在肺癌治疗中临床应用广泛，但仍存在一些内在因素限制其疗效。例如肺癌固有的放射抵抗导致许多恶性肿瘤患者对放疗反应不佳^[47⇓⇓⇓-51]^，而肿瘤乏氧、快速的DNA损伤修复，尤其是肿瘤的抑制性TME，导致了放疗的局限性和肿瘤的高复发率和高转移率^[52]^。已经证明工程化外泌体可重塑肺癌患者抑制性TME，因此有望通过工程化外泌体提高肺癌放疗疗效，起到协同抗肿瘤的目的。一项研究^[52]^中，将PD-L1抗体和DNA损伤修复抑制剂负载在M1型巨噬细胞来源的工程化外泌体上，其可以增强荷瘤（小鼠Lewis肺癌细胞）小鼠对放疗的敏感性。机制上考虑M1型巨噬细胞来源的工程化外泌体膜内部表达的过氧化氢酶可以有效缓解TME内的缺氧，同时加大DNA损伤。此外，先前封装在M1型巨噬细胞来源的工程化外泌体中的盘状结构域受体（discoidin domain receptor, DDR）抑制剂可以显著限制DNA损伤修复。M1型巨噬细胞来源的工程化外泌体可将巨噬细胞从M2表型极化为M1表型，其表面合成的抗PD-L1纳米抗体可以终止T细胞的免疫抑制^[52]^。

## 5 总结与展望

工程化外泌体在肺癌的化疗、TME的调控、基因治疗、放疗等方面可发挥重要作用，有望成为突破肺癌治疗瓶颈的手段，但其临床转化仍然面临诸多的问题和挑战：包括工程化外泌体的异质性，经过改造是否会引起外泌体初始生物性的变化，如何标准化批量生产、储存等。
